# Tumor-associated collagen signatures: pushing tumor boundaries

**DOI:** 10.1186/s40170-020-00221-w

**Published:** 2020-07-02

**Authors:** Elizabeth A. Brett, Matthias A. Sauter, Hans-Günther Machens, Dominik Duscher

**Affiliations:** Department of Plastic and Hand Surgery, Technical University Munich, Ismaninger Strasse 22, 81675 Munich, Germany

**Keywords:** Tumor-associated collagen signatures, TACS, Breast cancer, MDA-MB-231, Oncogenic, Metastasis, Collagen, Tumor

## Abstract

In 2006, a new model of invasive breast tumor emerged and, since 2011, is gaining recognition and research momentum. “Tumor-associated collagen signatures” describe 3 distinct layers of collagen which radiate outward in shells from the main body of the tumor. The outermost layer (TACS3) features branches of collagen radiating away from the tumor, 90° perpendicular to the tumor surface. TACS3 increases tumor span and correlates directly with metastasis, though presently difficult to detect in breast tissue. TACS is an emerging model but has been validated by multiple labs in vitro and in vivo, specifically for breast cancer prognostics. Newly recognized and accepted tumor borders will impact both R0 resections and downstream surgical reconstruction. This review aims to comprehensively introduce and connect the ranging literature on linearized collagen of invasive tumor borders. Using PubMed keyword searches containing “aligned,” “linear,” “oriented,” and “organized,” we have gathered the studies on TACS, integrated the concept into the clinic, and projected future platforms.

## Introduction

Understanding invasive breast tumor maturation has been the focus of breast cancer research for decades. Since 2006, there has been growing evidence of a previously unrecognized tumor feature which correlates with decreased patient survival rates. Tumor-associated collagen signatures (TACS) is a system which classifies three different collagen shells of the invasive breast tumor. Importantly, the outermost layer (TACS3) is defined by linear branches of collagen growing perpendicularly away from invasive breast tumors [1]. Within breast cancer literature, words like “aligned,” “oriented,” “organized,” and “linear” can be found referring to a specific extracellular matrix (ECM) pattern of the invasive breast tumors [2–4]. Clinically, there is a well-established link between the collagen architecture of the primary tumor and prognosis. In both canine and human mammary carcinomas (ductal and lobular), tumor borders with perpendicular protrusions of collagen correlated with metastasis, and TACS3-positive patients had statistically lower disease-free survival. Lymphatic and vascular invasion and increased syndecan-1 presence were also correlated with TACS3 in biopsy samples [5, 6]. While TACS is not a perfect diagnostic tool for other tumor features like hormone receptor status, it is explained in the later sections of this review that TACS3 studies largely feature triple-negative breast cancer cells, thereby removing ER, PR, and HER2 from the diagnostic/therapeutic options.

The aims of this literature review are to collect existing studies on TACS, assess what TACS means for the clinic, and consider future directions.

## Tumor-associated collagen signatures

**Fig. 1:**
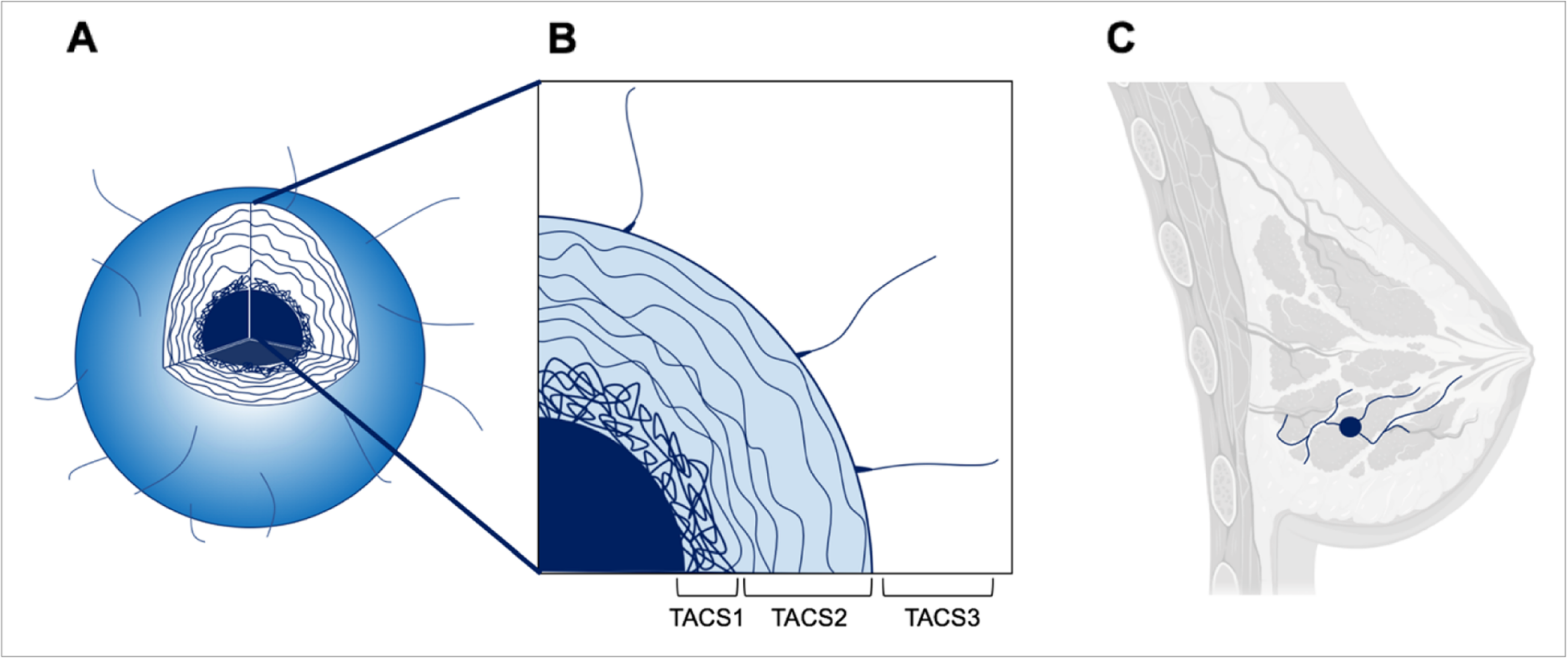
**a** Schematic of a 3-dimensional tumor with quadrant missing, to highlight the location of TACS layers. **b** Zoomed in breakdown of TACS layers from internal to external, 1–3. Specifically, TACS1: increased collagen deposition around the tumor, TACS2: alignment of layers around the tumor in a spherical manner, TACS3: development of perpendicular tracts of collagen away from the tumor. **c** Hypothetical application of TACS3 in co-opting normal breast structure for invasion and metastasis. Image made using biorender.com

In 2006, tumor collagen heterogeneity was described in the breast tumor using a tri-part system called “tumor-associated collagen signatures” or TACS. The classification breaks the tumor collagen into three zones, each of them physically distinct from one another [1]. TACS1 describes the inside layer of a densely packed collagen. TACS2 is a series of spheroidal shells around the TACS1 layer. TACS3 is defined by linear outgrowths of collagen leading from the tumor into breast parenchyma (Fig. 1). Many research papers which examine TACS are corollary in nature, with a large focus on describing the presence and prognostic value of TACS3. Our group recently published the linear formation of collagen in vitro by a breast cancer cell co-culture, which marks the first TACS publication to have a laboratory intervention experiment pertaining to linearized ECM of breast cancer [7]. TACS is largely described in breast tumors; however, orderly collagen formation is also seen in malignant ovarian tumors, with one study showing “increased normality” of collagen deposition [8]. While “collagen alterations” are seen as aligned waves of ECM in advanced ovarian tumors [9], no classification such as TACS has been adopted for this tumor or any other.

## TACS detection

Second-harmonic generation (SHG) microscopy is a non-linear microscopy technique which has been developed to enhance fibrosis research. The microscope structure is similar to that of an inverted light microscope and functions to analyze excised tissue en bloc. Briefly, a high-energy laser penetrates the sample, and when it meets the collagenous structures of specific orientation, the incident ray emitting from the sample is doubled in frequency. The ray is detected, interpreted through a series of formulae, and given a numerical value corresponding to isotropy/anisotropy [10]. Specifically, SHG can provide in-depth detail on fibrillar collagen and is useful in fibrosis, tumor, and connective tissue disorder research and diagnostics [11]. Limitations of the technique are the penetration depth, which is dependent on the scattering ability of the tissue (usually on the order of hundreds of micrometers). Similarly, it does not provide contrast for cells and is only highly specific for fibrillar collagen. While a low-cost version of a SHG microscope has been created, it still ranges around USD 40,000 [12]. Alternatives exist, for instance, spatial light interference microscopy (SLIM) has demonstrated better TACS3 detection along with better discernment of the tumor border, due to SLIM ability to measure linear and also non-linear collagen [3, 13]. The value of SHG may be in the binary confirmation of the presence/absence of linear collagen, as seen in past research which visualized the TACS3 collagen pattern in intact, unfixed human mammary gland samples [10]. For instance, SHG very successfully images the uterine wall tissue which is known for its linearity [14]. TACS is detectable using strategic histological sections made transversely at the tumor border. Recently, TACS3 at the tumor border of invasive breast tumors have been easily identified using classic hematoxylin and eosin staining [7]. However, this stain is only specific for collagen, not collagen isoforms. The same study used a parallel in vitro collagen deposition study and mass spectrometry to make an educated guess at which the isoform of collagen is highest in the TACS3 signatures. The proteome analysis of in vitro collagen showed collagen VI, which was seen using immunohistochemistry to be highly present in TACS3 of human tumor biopsy samples [7]. This represents the first reported biochemical target of TACS3. While more corroborative research needs to be published, visualizing collagen VI could represent a logical workaround to easy TACS3 detection, without having to source SHG.

## TACS in vitro

Many experiments in vitro assay show the preferential formation of lines/striated structures by non-cancer cells and triple-negative breast cancer cells. For instance, MDA-MB-231 cells cultured in two points opposite of each other over 10 days showed a reorganization of the collagen gel in between, from random formation to a linear pattern [15].. Linear manipulation of collagen gels is done by MDA-MB-231 cells, and a recent study also shows that linear de novo formation of collagen by a co-culture featuring MDA-MB-231 cells occurs. Our group recently uncovered an axis used by a trio of cells to deposit linear collagen type VI. Specifically, MDA-MB-231 cells and adipose-derived stem cells cultured in a juxtacrine manner produce high levels of CCL5. The CCL5 goes on to influence neighboring fibroblasts in a paracrine manner, which then produce linear collagen VI, revealed by decellularization of the culture after 7 days. When CCL5 is blocked, linear collagen deposition is prohibited and a randomly arranged layer of ECM is produced. Moreover, when MDA-MB-231 cells are reseeded on the linear matrix, they show high levels of proliferation and formation into road-like structures, the type of organized invasive behavior seen in enhanced metastatic abilities [7]. Velez et al. mirror these findings, but with “collagen-induced network phenotypes” using MDA-MB-231 cells in collagen gels. The research showed faster migration of cells on high-density gels, formation of linear cell networks stimulated by matrix architecture, and an increase in integrin β1 expression of migrating cells [16]. Together, these works show the feasibility of in vitro investigations on linear collagen of invasive breast cancer.

A 2016 publication features a novel in vitro arrangement where Matrigels of two different densities were interfaced. The less-dense 2-mg/ml gel contained MDA-MB-231 cells, and it was seen that these cells generated linear outgrowths which allowed invasion into the more dense 10 mg/ml gel [2]. In 2019, a study was run on the supportive role of non-cancer cells in guiding cancer cell protrusions into controlled 3D environments in vitro. The novel models involved tumor spheroids containing cancer-associated fibroblasts (CAFs) and cancer cells in a 1.9-mg/ml collagen type 1 solution. Cancer cells (pancreatic, lung adenocarcinoma) via integrin α5β1, bind to fibrillary fibronectin on the CAF membrane which provides a platform for migration [17]. Critically, it has been seen in breast tumor spheroids that CAFs are necessary for the secretion of ECM onto polycaprolactone scaffolds, which were later decellularized and seeded with MDA-MB-231 to create tumoroids [18]. This is yet another study using decellularization that shows the power of fibroblasts in the creation of breast cancer ECM.

## TACS in vivo

The hypothetical clinical role of TACS3 was substantiated in 2011. Not only was the TACS3 linear collagen presentation detectable on 4 μm histology sections across 196 human breast tumor samples, but a worse prognosis followed TACS3 biopsy presence [5]. Considering the prognostic value of this work, a machine learning model was developed to catalog and measure TACS3 in almost 200 biopsy samples and was found to be as accurate as three independent human observers as a structural biomarker in breast cancer histopathology [19].

**Fig.2:**
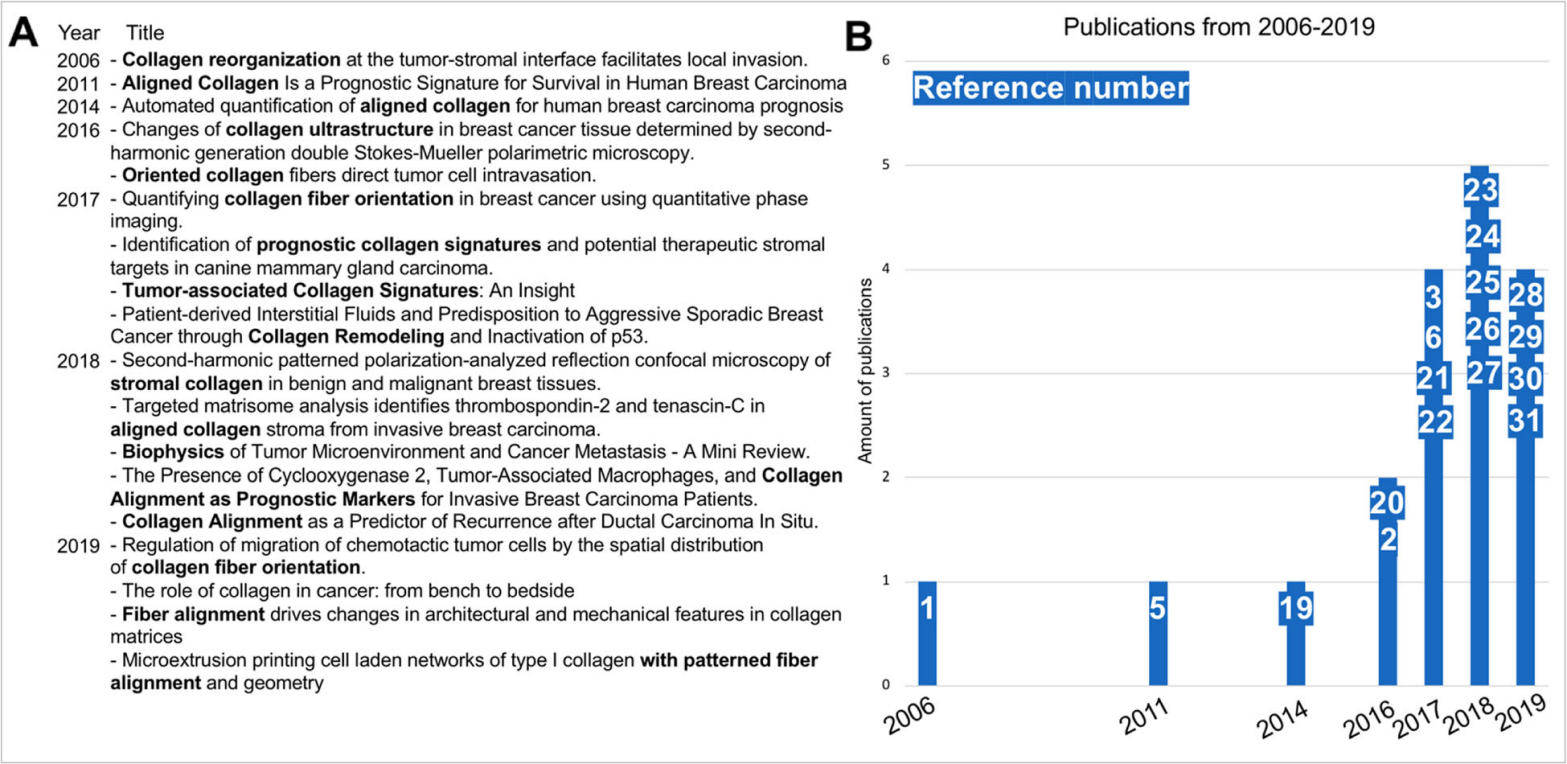
**a** List of publications resulting from PubMed keyword search using “linear collagen” AND “breast cancer,” “tumor associated collagen signatures” AND “breast,” “oriented collagen” AND “metastatic.” **b** Chronological graph showing an upgoing trend of publications featuring the keywords in **a**. White font on blue background denotes reference number, corresponding top to bottom as in the text of **a**

The studies resulting from a PubMed keyword search featuring “linear collagen” AND “breast cancer,” “tumor associated collagen signatures” AND “breast,” “oriented collagen” AND “metastatic” pertain to aligned collagen patterns at the breast tumor boundary [1–3, 5, 6, 19–31] (Fig. 2). The upward trend of the number of these publications highlights the momentum which the field gains annually. The collective goal of these publications is currently to establish TACS as a legitimate, overlooked tumor feature and to adopt TACS as a prognostic histopathology tool.

## Where to go from here?

The linearized outer coating of the invasive breast tumor, characterized by TACS3, bestows a range of pro-metastatic features [2, 5, 32]. There is precedent for a full investigation into the diagnostic/therapeutic impact of TACS3. While past in vitro literature focuses largely on collagen hydrogels, biasing the models to collagen type 1, the work from 2019 to 2020 shows the power of de novo collagen creation and decellularization, marking a huge step in the right direction of unbiased ECM study [7, 18]. The research of TACS3 in vivo will be greatly boosted by further work on the proteomic composition and novel target identification of TACS3 structures. This will allow the field to step away from SHG [10] and towards specialized staining which can inform tumor progression. The finding of collagen VI at the tumor border [7, 33, 34] is especially interesting given the nature of collagen VI breakdown products. Endotrophin (ETP) is an enzymatically generated fragment of the C5 residue from the α3 chain of collagen VI. It is automatically cleaved from the chain once the chain has left the cell body, making collagen VI both a structural and signaling protein [35]. ETP is in such high quantities in breast cancer patients that it is detectable in the blood [36] and acts as a pro-fibrotic, pro-EMT chemoattractant. Similarly, ETP has shown to increase patient resistance to cisplatin, and when blocked with antibodies in a mouse model, tumor growth is limited alongside increased sensitivity to chemotherapy [37]. An unanswered question of breast cancer ECM research, and likely the next most important, is the biomechanical properties of the collagen. There are no studies on the stiffness of collagen or viscoelasticity of cells interacting with the matrix, which represent large knowledge gaps in the prooncogenic nature of TACS. Conceptually, it is important to note that once TACS3 structures are present at the tumor border, it is likely that the tumor has already metastasized [5]. Therefore, studies should be focused on intervention therapy, to prevent the tumor from building the structures in the first place. The role of CCL5 has been described by our group in the formation of linearized breast cancer collagen, and while there is more confirmatory work to be done, blocking CCL5 presents a logical first step towards the goal of limiting triple-negative breast tumor evolution.

## Conclusion

As new research pertaining to tumor architecture emerges, it stands to inform oncologic management of the tumor, histopathological criteria, and surgical management. Existing literature across many labs reveal the in vivo TACS model to be reproducible and credible, and in vitro work to be informative and accurate. The trend of publications referenced herein highlights an increased focus on this developing model. A full understanding of TACS will further galvanize prognostics and potentially change the course of therapeutics and surgical practices. Perhaps the most impactful arm of research will be the preventative pharmacological studies, which should aim to limit TACS3 formation altogether.

## Data Availability

Not applicable
